# MAGUS: machine learning and graph theory assisted universal structure searcher

**DOI:** 10.1093/nsr/nwad128

**Published:** 2023-05-08

**Authors:** Junjie Wang, Hao Gao, Yu Han, Chi Ding, Shuning Pan, Yong Wang, Qiuhan Jia, Hui-Tian Wang, Dingyu Xing, Jian Sun

**Affiliations:** National Laboratory of Solid State Microstructures, School of Physics and Collaborative Innovation Center of Advanced Microstructures, Nanjing University, Nanjing 210093, China; National Laboratory of Solid State Microstructures, School of Physics and Collaborative Innovation Center of Advanced Microstructures, Nanjing University, Nanjing 210093, China; National Laboratory of Solid State Microstructures, School of Physics and Collaborative Innovation Center of Advanced Microstructures, Nanjing University, Nanjing 210093, China; National Laboratory of Solid State Microstructures, School of Physics and Collaborative Innovation Center of Advanced Microstructures, Nanjing University, Nanjing 210093, China; National Laboratory of Solid State Microstructures, School of Physics and Collaborative Innovation Center of Advanced Microstructures, Nanjing University, Nanjing 210093, China; National Laboratory of Solid State Microstructures, School of Physics and Collaborative Innovation Center of Advanced Microstructures, Nanjing University, Nanjing 210093, China; National Laboratory of Solid State Microstructures, School of Physics and Collaborative Innovation Center of Advanced Microstructures, Nanjing University, Nanjing 210093, China; National Laboratory of Solid State Microstructures, School of Physics and Collaborative Innovation Center of Advanced Microstructures, Nanjing University, Nanjing 210093, China; National Laboratory of Solid State Microstructures, School of Physics and Collaborative Innovation Center of Advanced Microstructures, Nanjing University, Nanjing 210093, China; National Laboratory of Solid State Microstructures, School of Physics and Collaborative Innovation Center of Advanced Microstructures, Nanjing University, Nanjing 210093, China

**Keywords:** crystal structure searching, materials design, *ab-initio* calculations, density functional theory, high-pressure phase transition

## Abstract

Crystal structure predictions based on first-principles calculations have gained great success in materials science and solid state physics. However, the remaining challenges still limit their applications in systems with a large number of atoms, especially the complexity of conformational space and the cost of local optimizations for big systems. Here, we introduce a crystal structure prediction method, MAGUS, based on the evolutionary algorithm, which addresses the above challenges with machine learning and graph theory. Techniques used in the program are summarized in detail and benchmark tests are provided. With intensive tests, we demonstrate that on-the-fly machine-learning potentials can be used to significantly reduce the number of expensive first-principles calculations, and the crystal decomposition based on graph theory can efficiently decrease the required configurations in order to find the target structures. We also summarized the representative applications of this method on several research topics, including unexpected compounds in the interior of planets and their exotic states at high pressure and high temperature (superionic, plastic, partially diffusive state, etc.); new functional materials (superhard, high-energy-density, superconducting, photoelectric materials), etc. These successful applications demonstrated that MAGUS code can help to accelerate the discovery of interesting materials and phenomena, as well as the significant value of crystal structure predictions in general.

## Teaser

Scientists developed a machine learning and graph theory assisted crystal structure prediction method (MAGUS), which can be used to accelerate the discovery of interesting materials and surprising physical phenomena.

## INTRODUCTION

Crystal structures are the starting points and basis of materials research, many of the studies on materials start with ‘what does it look like?’ Another important reason is that, with known crystal structures, it is possible nowadays to predict many physical and chemical properties by first-principles (or so-called *ab-initio*) calculations based on quantum mechanics. Hence, determining the crystal structure of materials is always a crucial problem in many research fields. For a long time, experimental methods such as X-ray powder diffraction (XRD) and neutron scattering, have been the main ways to determine crystal structures, while computational methods have become another viable option, recently, due to the enormous developments of computational hardware and software, such as supercomputers and computational algorithms (especially density functional theory [1,2]). Crystal structure prediction (CSP), which aims to find stable or metastable structures under given conditions (pressure or temperature), is becoming an effective tool for discovering new materials and exploring the phase space [3–6]. It can automatically identify unknown structures in experiments with little prior information, sometimes with only the chemical compositions, which used to be considered impossible.

In the view of scientific computing, CSP can be regarded as a global optimization problem on a complex potential energy surface (PES), which can be split into two sub-questions: how to estimate the stabilities of the sampled structures and how to sample the configuration space. The past decades have witnessed the rapid development of computing power and improvements in algorithms that help address these two challenges. For the first challenge, first-principles computational methods are common choices to calculate the energy or the enthalpy and determine the thermodynamic stability of the candidate structures. For the second challenge, many algorithms (always combined with structure relaxations) have been proposed and integrated into the corresponding codes. A large number of successful cases have emerged in various research fields including physics, chemistry, materials science, etc. To list only a few of them: AIRSS [7] uses the random structure searching method and determined the structure of SiH_4_ under high pressure [8]; Doll, Schön, and Jansen applied simulated annealing and succeed in determining the structures of lithium fluoride [9] and boron nitride [10]; Gödecker came up with the minima hopping method [11] and determined the structure of silicon clusters [12]; USPEX [13,14] used evolutional algorithm (EA) and found many unexpected structures including transparent dense sodium [15]; CALYPSO [16] used particle swarm optimization (PSO) and predicted the high-pressure phase of lithium [17]. More, but not all, examples can be seen in Table 1 [7–11,13–72]. However, despite all the success stories, the accessible size of the system is still limited by two basic difficulties: the number of local minima grows exponentially with the degrees of freedom (or the number of atoms in the system), and the cost of *ab-initio* calculations in local optimizations grows rapidly with the number of atoms. To alleviate these challenges, we make use of graph theory and machine-learning potential to develop the code called Machine learning And Graph theory assisted Universal structure Searcher (MAGUS).

**Table 1.** Some crystal structure prediction software and part of their applications.

Software/Author	Sample method	Part of application
Goedeckor *et al*. [11,18]	Minima hopping	Cold compressed graphite [19]
		High-pressure disilane [20]
		LiAlH_4_ [21]
Jensen *et al.* [9]	Simulated annealing	Lithium fluoride [9]
		Boron nitride [10]
Reuter *et al.* [22]	Simulated annealing	IrO_2_/RuO_2_ surface [22]
AIRSS [7,8]	Random search	SiH_4_ [8]
		Phase III hydrogen [23]
		Ionic phases of ammonia [24]
SYDSS [25]	Random search	H_2_O-NaCl [25]
		C-O [25]
		Cl-F [26]
Genarris [27,28]/GATor [29]	Random search/Evolutionary algorithm	Chiral arene [30]
USPEX [13,14]	Evolutionary algorithm	Gamma-boron [31]
		Transparent dense sodium [15]
		Na-Cl [32]
XtalOpt [33]	Evolutionary algorithm	Sodium polyhydrides [34]
		High-pressure ices [35]
		Condensed astatine [36]
GASP [37]	Evolutionary algorithm	Li-Be alloys [38]
		Europium [39]
		Li-Si [40]
MAISE [41]	Evolutionary algorithm	Fe-B [42]
		Ca-B [43]
		NaSn_2_ [44]
MUSE [45]	Evolutionary algorithm	NbSe_2_ [46]
		High-pressure gold [165]
		IrB_4_ [48]
EVO [49]	Evolutionary algorithm	Li_13_Si_4_ [50]
IM^2^ODE [51]	Differential evolution	Low bandgap TiO_2_ [52]
		Hybrid sp2-p3 carbon [53]
		2D SiS [54]
SGO [55]	Differential evolution	
AGA [56]	Adaptive genetic algorithm	Zr-Co [57]
		MgO-SiO_2_ [58]
		LiNiB [59]
CALYPSO [16]	Particle swarm optimization/Artificial bee colony	High-pressure Li [17]
		XeFe_3_ [60]
		LaH_10_ [61]
BEACON [62]/ICE- BEACON [63]	Bayesian optimization	Cu_15_ [62]
		Ta_2_O_5_ [62]
		CuNi clusters [63]
GOFEE [64]	Bayesian optimization + genetic algorithm	PAH on graphene [65]
		C_24_ clusters [66]
		Carbon clusters on Ir(111) [66]
CrySPY [67]	Bayesian optimization + genetic algorithm	Y_2_Co_17_ [68]
		Al_2_O_3_ [68]
		GaAs [68]
MAGUS [69,70]	Evolutionary algorithm with machine learning and graph theory	WN_6_ [69]
		HeH_2_O [71]
		SiO_2_ [72]

A crystal structure is always represented by its lattice parameters as well as the type and the coordinate of each atom. However, sometimes the atomic positions are not the best way to describe crystals, and the topological properties of crystals can be helpful in this regard. In graph theory, crystals can be interpreted as graphs by considering atoms as nodes and bonds as edges. Graph theory is not a new idea in crystallography and has been used for a variety of purposes including structure generation. For example, Shi *et al.* [73] proposed a method using quotient graphs to stochastically generate crystal structures with specified connectivity and found a series of complex *sp*^3^ carbon polymorphs with large band gaps [74]. Bushlanov *et al.* [75] used a generator based on topological databases containing idealized periodic nets from known crystal structures [76,77] to produce random structures in their evolutionary structure searching. The topological generator can sample more ordered structures with lower energies than the symmetric random generator, which results in significant performance improvement. Deringer *et al.* [78] used the modularity optimization technique to decompose networks into clusters and build random structures using these clusters for prediction in their *ab-initio* random structure searching (AIRSS) [7]. This approach can simplify configuration space and successfully find low-energy hypothetical allotropes of boron and phosphorus [78,79]. These previous works showed the power of graph theory in reducing the complexity of searching space and proved that it can accelerate the searching process.

On the other hand, machine-learning potential (MLP) has been a hot topic in computational physics and materials simulations [80] in recent years because a well-trained MLP can achieve an accuracy comparable to density functional theory (DFT) with significantly less computational cost. Thus, it can help us explore much larger systems that are very hard for DFT. However, we usually do not have a well-trained MLP before most new cases of crystal structure predictions. A common approach is active learning [81], which trains an on-the-fly MLP from scratch during the prediction processes. Tong *et al.* [82] trained Gaussian approximation potentials [83] to perform the local optimization and proposed a new global minimum structure for the B84 cluster. Deringer *et al.* [78]. developed an approach called GAP-RSS, whose effectiveness can be proved by its success in many systems including carbon [84], phosphorus [78], and boron [85,86]. Kolsbjerg *et al*. proposed two schemes, one is to use neural network potentials as an agent of the DFT in relaxation but retain DFT single-point calculations in every generation and find a Pt13 nanoparticle on a MgO substrate [87], and the other is to adopt the idea of Bayesian optimization to select the structures to be added [64] which are similar to our previous work [69], and significant acceleration was achieved compared to the pure first-principles process based evolutionary algorithm in surface reconstruction tasks. Podryabinkin *et al.* [88] have combined moment tensor potential [89,90] with USPEX [13]. They reproduced the known results in the test systems but with much less computation, and successfully predicted an unknown structure of boron with 54 atoms in the unit cell. From the above, we can see the effect of MLP on the acceleration of local optimization.

Since these two approaches are compatible, we combine them with our current version of MAGUS based on the evolutionary algorithm to enhance crystal structure predictions. The paper is outlined as follows: in the Design and Techniques Section, we introduce the current version of our MAGUS code starting from EA, and how we leverage graph theory and machine learning for acceleration. In the Performance Tests Section, we first provide the competitive effectiveness of our basic version. The performance improvements brought by graph theory and machine learning is tested in many different systems. In the Applications Section, we introduce some representative achievements in different topics, including compounds in the interior of planets; new functional materials (superhard, high-energy-density, superconducting, photoelectric materials); phase transitions and properties adjustment of quantum materials under high pressure, etc.

## DESIGN AND TECHNIQUES OF THE MAGUS CODE

**Figure 1.**
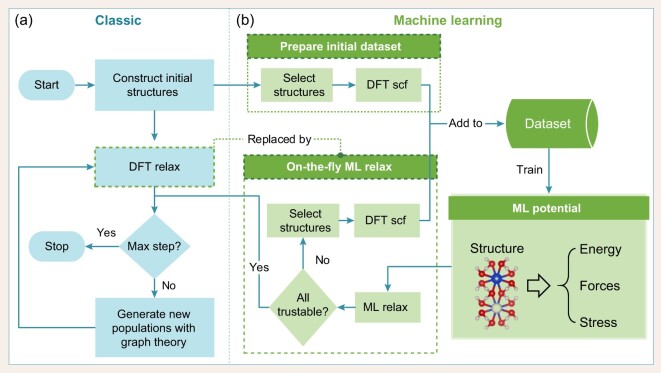
Workflow of MAGUS, which includes (a) classic evolutional algorithm and (b) machine-learning crystal structure predictions.

As shown in Fig. 1, The workflow of the MAGUS code can be divided into two parts: the classic evolutionary algorithm and the machine learning part. During the structure evolution, we used graph theory to decompose the structures. In the following, we explain these three parts in detail.

### Classic evolutionary algorithm

**Figure 2.**
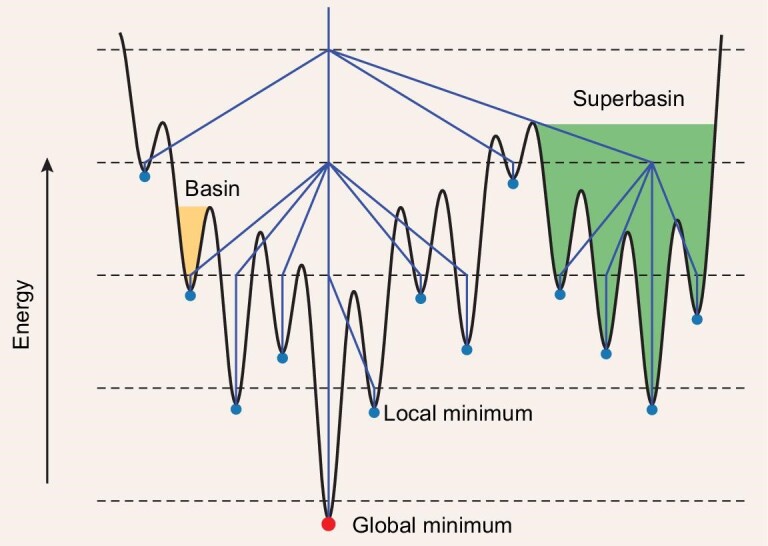
A schematic illustration of a one-dimensional potential energy function and the corresponding disconnectivity graph. The blue points are the local minima and the red is the global minimum. The structures can be relaxed to the same local minimum comprising a basin. And this concept can be extended to ‘superbasin’ in which any two minima can be interconverted without exceeding the given potential energy. A disconnectivity graph is a tree graph that shows the number and distribution of superbasins at different energy levels.

The potential energy landscape of a crystal system can be rugged and funnel-like [91] with a lot of local minima separated by barriers. A typical sketch map with disconnectivity graph [92] of the 1D condition is shown in Fig 2. To find the only one global minimum among the local minima, the PES should be adequately explored. Many crystal structure searching methods are applied, such as random search, simulated annealing, minima hopping, meta dynamics, particle swarm optimization, and evolutional algorithm as shown in Table 1. In our code, the evolutional algorithm was adopted for its flexibility to be combined with other theoretical tools.

Evolutional algorithm, which draws from the biological evolution mechanism in nature, is common in carrying out efficient global searches. To apply it to crystal structure predictions, we regard a single candidate structure as an individual and a set of them as a population. Just like in nature, they generate new populations called offspring by crossover and mutation, and the individuals with good fitness are superior to survive. Combined with local optimization, the evolutional algorithm has been proven to be a powerful algorithm in crystal structure predictions [93].

**Figure 3.**
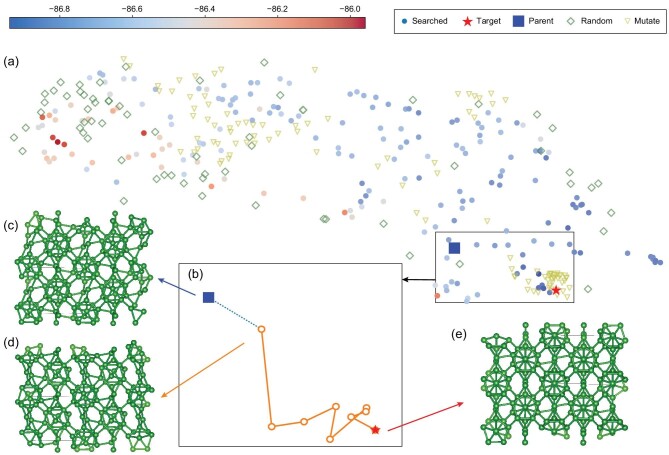
The 2D energy landscape visualized by UMAP. (a) The circles are all the relaxed structures explored by one MAGUS searching for gamma boron. The enthalpy of each structure is represented by its color as shown in the top color bar. We can see the top left corner region has relatively higher enthalpy and that of the region near the target marked by the pentagram is lower. The green diamonds and the yellow triangles are the relaxed structures generated by random and mutating the structure (c) marked by the blue square, respectively; (b) a path from (c) to (e). Because the unrelaxed high-energy structures may not be on the manifold, only the last 10 steps are drawn; (c) a good structure with relatively lower enthalpy and so has a high chance of being chosen as parents. The mutations are all based on this structure for convenience; (d) the structure of mutated (c); (e) the structure of gamma boron.

The reason why the evolutional algorithm together with relaxation work can be roughly understood from Fig 2. Structures that fall into basins are relaxed to the corresponding local minima. Since low-energy structures tend to gather in a superbasin with a relatively low energy barrier between each other, a small perturbation to a structure may lead it to fall into a nearby basin and relax into another local minimum. In this way, we can efficiently explore the superbasin. Considering the possibility that the global minimum is far away from the known structures or the superbasins are far away from each other, random structures are also required to thoroughly sample the PES. To further explain how it works in real systems, we take the example of searching for gamma boron. We calculate the Smooth Overlap of Atomic Positions (SOAP) [94] descriptor of all the relaxed structures we explored during the search process and use UMAP [95] to embed it into 2 dimensions to visualize the energy landscape in Fig 3. In general, the distance between two points can be regarded as the similarity between two corresponding structures: the more similar two structures are, the closer they are to each other. The energies and forces are calculated with GAP [83] potential for boron [85] with the Python library pyquip [96]. The global minimum gamma boron with space group *Pnnm* is marked by a red pentagram as shown in Fig. 3b. Then we separately generated and relaxed 100 structures by random generation and by mutating a low-enthalpy structure shown in Fig. 3c with space group *P2_1_/c* (blue square), respectively, and compared their distribution. It can be seen that very few random structures after optimization (green diamond) fall into the area near the gamma boron and many of them landed at the high energy zone. (See online supplementary material for a color version of this figure.) On the contrary, a lot of mutated structures are very close to the target structure after optimization, which means the evolutional algorithm can be biased once good structures are given and focus on the more promising areas. A path from the parent structure to the target can be seen in Fig. 3b. The *P2_1_/c* boron is initially mutated to a structure shown in Fig. 3d and jumps out of the local minimum and successfully relaxed to the target.

In detail, the workflow of our MAGUS code can be seen in Fig 1. It first generates a set of structures and reads given seeds to form the initial population. Some tricks described later can be used to improve the quality of the random structures. Next, the algorithm iterates the following steps until reaching the settled maximum number of generations. In each generation, the structures in the population are firstly relaxed with DFT or any potential depending on the requirement. After that, repetitive structures are removed in order to keep diversity. Then the remaining structures are selected to create offspring by crossover and mutation based on fitness. These offspring as well as some new random structures compose the new population.

Next, we provide a more detailed description of all the steps of our program in the following.

#### Fitness

In the evolutional algorithm, one of the initial steps is to determine the fitness function, as it guides the direction of the search. Fitness evaluates the quality of a single structure, the structures with higher fitness should have a higher probability to be chosen as parents for procreation. Depending on the search task, fitness can be defined differently.

For searches with given compositions, enthalpy per atom is a good choice as the most stable structure should have the lowest value. However, it should be noted that the structure with the lowest enthalpy found during a search for a given composition may not be stable and could decompose in nature. The stabilities of structures with various stoichiometries can be determined by the convex hull of the enthalpy per atom according to the fraction of each chemical composition. Structures on the convex hull are stable, while those above the hull may be metastable. Therefore, if the chemical composition is uncertain, the composition space should also be searched [97]. In this condition, we can take the energy above from the current convex hull as fitness. The uncertainty of the target property can also be taken into account with Bayesian optimization [69].

Fitness can also be designed to search for materials with desired properties like hardness [69,98–100] and bandgap [52]. We cannot only optimize these objectives, otherwise the resulting structures may be unstable and cannot be synthesized. Therefore, these objectives often need to be optimized together with enthalpy (or enthalpy above the convex hull in variable composition search) as much as possible, but these variables often cannot be simultaneously optimized to their fullest potential. Such a multi-target optimization problem can be solved using Pareto optimization. If a solution B is no worse than another solution A in all target properties, we say that A is Pareto-dominated by B. We can use the number of structures that are Pareto-dominated as the fitness in this case.

#### Generate initial structures

A good starting population with high diversity and low energy can significantly speed up the search process. By placing some restrictions on the initial structure, such as the lattice parameters, minimum interatomic distances, and space group, which are provided by assumption or experiment, a purely random search can also discover stable structures. Therefore, having a good sample of structures is critical for creating the initial population and introducing random structures into the subsequent population.

##### Generate structures with a given space group.

For large systems with high intrinsic dimensionality [101,102], random sampling without constraints always leads to similar disordered structures with relatively high energy. On the other hand, symmetry is usually prevalent in the extended compounds and only very few inorganic crystals belong to the *P1* [103]. Generating structures with a given space group can help to alleviate these two problems. Here, we follow a process similar to RandSpg [104]. For a crystal with a given space group and the number of atoms, we first try to get a pool of possible Wyckoff position combinations. A possible Wyckoff position combination should meet two requirements: each unique Wyckoff position (the Wyckoff position with fixed coordinates) can only be used no more than once; and for each atomic species, the sum of the multiplicities of its Wyckoff positions should equal its number of atoms. Once a series of combinations have been created, a lattice constrained by the space group will be generated. After these two steps, the program attempts to place atoms into the lattice based on the given Wyckoff position combination in sequence. If some atoms are closer than the given threshold, free variables in the Wyckoff position will be regenerated randomly until the distance limit is satisfied or the maximum number of attempted steps is reached. For structures other than 3D clusters, the process is similar but the 230 three-dimensional space group should be replaced by the corresponding groups, such as plane group or layer group for layer structure [105], point group for cluster, etc.

For molecular crystals [106], it is slightly more complicated. Because the molecules themselves may have their symmetry, simply putting it into Wyckoff position as a whole, like atoms, may break the target symmetry. A molecule can be put into a Wyckoff position with some specific orientation only if the Wyckoff site symmetry is a subgroup of the molecule's point group symmetry, so the validation of the Wyckoff position is determined as follows. First, we obtained all the symmetry operations of Wyckoff positions. If there is only one axis of symmetry, then check whether the molecule also has the same axis. The molecule can rotate arbitrarily as long as the two axes coincide. If there is more than one axis of symmetry, then calculate the angle between every pair of them and find the axes pairs with the same angle in the molecular. Take site symmetry *mm2* for example, it has a two-fold axis along the z-axis, and two reflection axes along the x-axis and y-axis. If the molecule also has a two-fold axis and two reflection axes and the angles between them are 90°, it can be put on the Wyckoff position with the two-fold axis along the z-axis. With these orientations’ constraints, the molecules can be put into a valid Wyckoff position like the process above.

##### Generate surface structures based on known bulk materials.

This is designed to generate the initial population of surfaces for the surface prediction module [105], which is inspired by some natural surface reconstruction processes such as dimers on diamond surfaces [107]. On a clean cleaved diamond surface, two dangling bonds form an additional bond to lower its surface energy, which is simulated by the random walk of atoms on the surface. In addition, appending or removing atoms at random is also applied, considering some reconstructions are adsorption-induced, and the formula of the surface structure is not always the same as its bulk form for satisfying the electron-counting rule. This method generates very similar structures which are less favorable in the evolutionary algorithm, so it is only used to supplement former methods in a surface prediction run.

##### Cell splitting.

Cell splitting is another way to reduce the degree of freedom of the system and generate more ordered structures [18,102,108]. Instead of generating a large cell directly, it generates a smaller subcell and replicates it to form a large cell. It is easy to see that such structures are more ordered, and thus have lower energy on average compared to normal random sampling [102]. If the stoichiometry of the system is not divisible by the number of subcells, the number of each atomic type in the subcell is rounded up and removes superfluous atoms after replication.

#### Variation operators

Variation operators are crucial components of any evolutionary algorithm as they dictate how the potential energy landscape is explored. A set of well-designed operators can significantly accelerate the search process. Depending on the number of parents involved, variation operators can be classified into two types: mutation involving a single parent and crossover involving two parents. MAGUS provides support for the following basic operators as well as any derived operators obtained by combining multiple basic operators.

##### Cut-and-splice (crossover).

**Figure 4.**
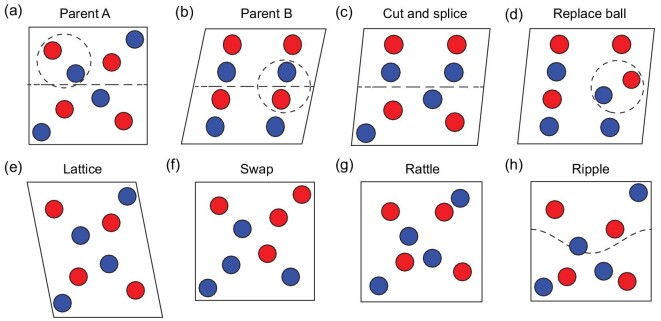
Examples of crossover and mutation. (a) Parent structure A for crossovers and mutations; (b) parent structure B for crossovers; (c and d) offspring structures of (a) and (b) by ‘cut-and-splice’ and ‘replace ball’ crossover; (e–h) offspring structures of (a) by ‘lattice’, ‘swap’, ‘rattle’ and ‘ripple’ mutation.

The real-space cut-and-splice operation was initially developed in clusters [109] and later extended to crystals [13]. As its name implies, it cuts two structures and splices them together. Figure 4c illustrates an example of how this operator works. First, two parents are selected at random to prepare for breeding, all atoms in each cell are translated by a random vector to increase randomness. For simplicity, but without loss of generality, we assume that the cut is parallel to the x-y plane. A fraction *k*, randomly chosen around 0.5, determines where to cut the two parents. The new lattice parameters are determined by using *k* to calculate the weighted average of the parents’ lattice parameters, and the positions of the new atoms are composed of two parts: atoms with fractional coordinates in the z-axis smaller than *k* from parent A and atoms with fractional coordinates in the z-axis larger than *k* from parent B.

##### Replace ball (crossover).

The cut-and-splice operator primarily utilizes fractional coordinates information and, therefore, the local configuration may undergo significant changes if the new lattice undergoes substantial changes. In contrast, the replace ball operator [55] chooses two balls at random points with the same random radius in the two parent structures and exchanges all atoms in them, as depicted in Fig. 4d. A random rotation may be applied to avoid biasing a given orientation. This operator can create two offspring, and we randomly select one of them.

##### Lattice strain (mutation).

The lattice strain mutation modifies the cell parameters by multiplying a strain matrix while keeping the fractional coordinates of the atoms fixed (as shown in Fig. 4e). The disturbance ${\epsilon }_i$ is sampled from a zero-centered normal distribution with a specified standard deviation.


(1) \begin{eqnarray*} &&\left( {a,b,c} \right) \to \left( {a^{\prime},b^{\prime},c^{\prime}} \right)\\ &&\qquad\quad = \left( {\begin{array}{@{}*{3}{c}@{}} {1 + {\epsilon }_1}&\,\,\,{\frac{{{\epsilon }_6}}{2}}&\,\,\,{\frac{{{\epsilon }_5}}{2}}\\ {\frac{{{\epsilon }_6}}{2}}&\,\,\,{1 + {\epsilon }_2}&\,\,\,{\frac{{{\epsilon }_4}}{2}}\\ {\frac{{{\epsilon }_5}}{2}}&\,\,\,{\frac{{{\epsilon }_4}}{2}}&\,\,\,{1 + {\epsilon }_3} \end{array}} \right)\left( {a,b,c} \right).\\ \end{eqnarray*}

##### Swap atoms (mutation).

The swap atoms mutation randomly selects an atom and then exchanges it with another atom with different chemical composition without changing the cell parameters (Fig. 4f). For molecules in molecular crystal structure searching or obtained by graph decomposition algorithms, any molecule with more than one atom can be selected for swapping, even if they have the same chemical composition.

##### Move atoms (mutation).

Random or purposeful movement of atoms is an intuitive way to introduce mutations into a configuration. Unlike swap atoms mutation, which can significantly alter the positions of atoms, move atoms mutation induces a displacement to atoms, resulting in a distorted structure. MAGUS now has four move atoms operators, namely, rattle mutation [110], ripple mutation [33], rotate motif mutation [3] and soft mode mutation [14,102]. The movement in the rattle mutation is independent and in the rest mutations are collective.

Rattle mutation repeats randomly, selecting an atom and moving it in a sphere with its original position as the center (Fig. 4g). Ripple mutation [33] adds a wave perpendicular to a randomly chosen axis to the structure (Fig. 4h). For demonstration purposes, we use the *z*-axis, and every atomic fractional coordinate along the *z*-axis moves based on its position in the x–y plane.


(2) \begin{eqnarray*} &&\left( {{f}_x,{f}_y,{f}_z} \right) \to \left( {{f}_x,{f}_y,{f}_z + \rho \cos\! \left( {2\pi \mu {f}_x} \right)}\right.\\ &&\quad \quad \quad \quad \ \ \ \ \times \left. {\cos \!\left( {2\pi \eta {f}_y} \right)} \right),\quad \mu \in Z,\eta \in Z.\\ \end{eqnarray*}

Rotate motif mutation randomly rotates a closely related component as a whole. It is typically used in molecular crystal searches where molecules are viewed as components. It can also be used in normal bulk systems combined with graph decomposition algorithms.

The above mutations move the atoms randomly, so the quality of offspring cannot be guaranteed. To address this issue, a special operator with a physical idea was proposed. Soft mode mutation displaces the atoms along the eigenvectors of a soft mode. This approach is effective because it is highly unlikely to encounter high barriers along a soft direction [111], and crossing low-energy barriers is more likely to achieve lower local minimums [112]. The main question of soft mode mutation is the Hessian matrix which is very expensive to calculate by DFT. A pairwise harmonic model is always used as an approximation and gives satisfactory results [14,102]. More accurate potential, such as the on-the-fly machine-learning potential, can also be used to construct the Hessian matrix.

##### Add/Remove atoms (mutation).

This operator alters the composition of configurations and was initially proposed by Lepeshkin *et al.* [113] to carry out a combined evolutionary search for clusters of varying formulas. It selects atoms for removal or addition based on a specific weight, with weakly bound atoms being more likely to be chosen. This ensures that good structures are spread across different compositions in cluster systems. In the surface prediction module [105] of MAGUS, this method is also utilized to distribute building blocks and decrease surface energy.

##### Symmetrization (mutation).

Symmetrization contains two ways of adding symmetry to a child structure, namely adding rotational symmetry and adding reflection symmetry. For the former purpose, this method firstly cut one parent into N pieces (N can be 2, 3, 4 and 6), and rotate one piece 360/N degrees around an axis parallel to the cutting plane which generates a child having N-fold rotational symmetry. For the latter purpose, this method cut one parent into two pieces and reflects one of them through the cutting plane. This method is designed to get structures into higher symmetry and is also applied in surface systems [105] in MAGUS, in which the symmetry axis and reflection plane are the same with their substrate rather than randomly chosen in cluster systems.

##### Structure repairing.

The above operators and their combinations may sometimes produce undesirable structures, such as incorrect formulas or too small interatomic distances. To address this, MAGUS includes a special operator that aims to repair such structures. First, we randomly remove atoms that are too close to each other until all interatomic distances meet the requirements. After that, we attempt to repair the formula by finding compositions that are close to the current one based on the type of search. If there are any atoms to be removed, we deleted them randomly. When adding a new atom, a random existing atom is chosen as the center, and the new atom is randomly placed within the sphere with a tolerance distance as the radius. This approach can improve the success rate compared to completely random placement.

#### Keep diversity

Maintaining population diversity is crucial in evolutionary algorithms to avoid solutions being biased towards a specific feasible space. Introducing randomness during structure generation and offspring creation is one approach, but it may not be enough since relaxed structures can still be similar and closely located in the search space due to the shape of the PES (Fig. 2). If the structures in the parent pool are highly similar or remain unchanged for a long time, they may easily fall into a local minimum. Thus, a niching method aimed at obtaining multiple basins is necessary.

##### Structure comparison.

One way to maintain diversity is by removing similar structures before adding them to the parent pool. However, this is not a simple task due to the various choices of the basis vectors, the ambiguous position of the origin, and the permutation invariance of the atomic order. To address these issues, MAGUS supports three methods based on crystal fingerprint, atomic environment descriptor, and point cloud registration, respectively.

The crystal fingerprint method extracts various properties of the structure, such as the number of atoms, chemical composition, unit cell volume, radial distribution functions, space group, Wyckoff information, and enthalpy. These fingerprints are rough and may result in many false positives, so some more accurate crystal fingerprints are constructed with pair distribution functions [91,101] and bond characterization matrices [114].

The atomic environment descriptor method [115] calculates the fingerprints of every atom instead of the entire crystal. This type of descriptor is related only to local atomic geometries within a cutoff radius, and therefore independent of the choice of lattice and origin position. With various definitions of the distance, we can compare all of the local environments belonging to two structures and generate a similarity matrix. The matrix can be then matched to a value by four different approaches as described in [116].

The point cloud registration method aims to find a direct one-to-one atom mapping between two structures [117,118]. The crystals to be compared are first standardized and Niggli-reduced to avoid the arbitrariness of the unit cell. The algorithm then finds all candidate transform matrices that matches two lattice vectors. If there exists a possible transformed cell and origin points that all atoms are close to their images, the two crystals are considered duplicates; otherwise, they are regarded as different.

##### Clustering.

To maintain a balance between diversity and quality in the search process, it is necessary to allow some good structures to survive in the next parent pool. The kept structures should be different from each other, and their number should be limited [14]. The distance between structures can be calculated by the first two methods mentioned above. With the definition of distance, we can apply clustering to the population and only keep the best structures in each cluster. By choosing an appropriate keep number, this algorithm can increase the quality of the population without compromising diversity. Additionally, clustering also affects the results of variation. For crossover operations, parent structures should come from the same cluster. Structures in the same cluster are usually in the same superbasin or funnel, making crossover operations between them easier to produce low-energy offspring. In contrast, offspring between structures from different superbasins are usually on a high barrier between superbasins [3,102].

### Graph theory enhanced configuration space exploration

In the idea of the evolutionary algorithm, the advantage ‘genes’ of parents should be preserved in the offspring. For crystal structures, the ‘genes’ are the local motifs since the properties are determined by the spatial arrangement of the atoms. If such excellent local structures are found, we should try to preserve them during the evolution process. For example, in the search for molecular crystals, the given molecule is an atomic arrangement that needs to be preserved. However, for a normal search without pre-defined molecules, it is not easy to determine these good motifs. In this case, decomposition methods based on graph theory are very helpful. Here we abstract a periodic crystal structure as a graph and use a community detection algorithm to do this job.

#### Convert crystal to quotient graph

The natural method to map a crystal structure to a graph is to treat each atom as a node and connect these nodes with an edge if the corresponding two atoms are covalently bonded. Since the crystal structure extends infinitely due to the periodic boundary condition, the induced graph also extends infinitely and is called the net graph (NG) here. Fortunately, the atoms and bonds in the different cells are equivalent, leading to the equivalent nodes and edges in the NG. Therefore, we can reduce the NG into a quotient graph (QG).

Let *G* be a graph. If *G* has edge set *E* and vertex set *V* and *R* is the equivalence relation induced by the partition, then the quotient graph has vertex set *V/R* and edge set $\{ ( {{{[ u ]}}_R,\ {{[ v ]}}_R} )\ |\ ( {u,\ v} )\ \in \ E( G )\} $. In our case, the equivalence relation *R* is induced by translational symmetry or *P1* symmetry. Note that there may be more than one edge between nodes ${[ u ]}_R$ and ${[ v ]}_R$, because an atom *A* can simultaneously bond with atom *B* and its equivalent atom *B′* in a different cell. Therefore, we need a notation to label the edges in order to distinguish them. An intuitive approach is to label edges with the relative cell coordination of the origin atoms. Specifically, we mark an atom at $( {{x}_i + l} )a$ as ${n}_i( l )$, where ${x}_i$ represents the fractional positions of the ith atom in the unit cell,${\rm{\ }}l$ denotes the coordination of the cell, and *a* is the basis vector. If an atom ${n}_i\!( {{l}_1} )$ is bonded with another atom ${n}_j\!( {{l}_2} )$, there will be a direct edge from ${v}_i$ to ${v}_j$ labeled as $l = {l}_1 - {l}_1$ in the derived quotient graph. Note that the edge remains unchanged with the opposite direction and a negative label vector. In other words, ${v}_i\mathop \to \limits^{\scriptscriptstyle\boldsymbol{l}} {v}_j$ is same as ${v}_j\mathop \to \limits^{\scriptscriptstyle - {\boldsymbol{l}}} {v}_i$. In summary, given a crystal with N atoms in the unit cell, we can obtain a labeled and directed QG(E, V) with E = $\{ {{v}_1,{v}_2, \ldots ,{v}_N} \}$ and V = $\{ {v}_i\mathop \to \limits^{\scriptscriptstyle\boldsymbol{l}} {v}_j,\ {v}_j\mathop \to \limits^{\scriptscriptstyle - {\boldsymbol{l}}} {v}_i|( {{n}_i( {l + {l}_0} ),\ {n}_j( {{l}_0} )} ) \in E( {NG} )\} $, where ${{\boldsymbol{l}}}_0$ is an arbitrary integer vector.

#### Dimensionality identification

In our previous work, we computed dimensionalities of crystal structures based on QG [119]. Let ${\boldsymbol{X}}$ be a component including all equivalent atoms${\rm{\ }}\{ {{n}_i(\bf 0 ),{\rm{\ }}{n}_i( {{{\boldsymbol{l}}}_1} ),{\rm{\ }}{n}_i( {{{\boldsymbol{l}}}_2} ), \ldots } \}$ connected in the NG and let ${\boldsymbol{l}}$ be a matrix whose rows are $\{ {{{\boldsymbol{l}}}_1,{{\boldsymbol{l}}}_2, \ldots } \}$, the dimension of ${\boldsymbol{X}}$ is then given by:


(3) \begin{eqnarray*} \dim \!\left( {\boldsymbol{X}} \right) = \dim \!\left( {\left\{ {{{\boldsymbol{l}}}_1,{{\boldsymbol{l}}}_2, \ldots } \right\}} \right) = {\rm{rank}}\left( {\boldsymbol{L}} \right). \end{eqnarray*}

It is evident that if a path exists between two equivalent atoms ${n}_i( {{{\boldsymbol{l}}}_1} )$ and ${n}_i( {{{\boldsymbol{l}}}_2} )$ in the NG, we can always find a closed chain that starts and ends at ${v}_i$ in the QG, and vice versa. For instance, consider the path ${n}_i( {{{\boldsymbol{l}}}_1} ) \to {n}_j( {{{\boldsymbol{l}}}_1} ) \to {n}_k( {{{\boldsymbol{l}}}_0} ) \to {n}_i( {{{\boldsymbol{l}}}_2} )$ between two atoms ${n}_i( {{{\boldsymbol{l}}}_1} )$ and ${n}_i( {{{\boldsymbol{l}}}_2} )$ in the NG, which is represented as ${v}_i\mathop \to \limits^0 {v}_j\mathop \to \limits^{{l}_1 - {l}_0} {v}_k\mathop \to \limits^{{l}_0 - {l}_2} {v}_i$ in the QG, and this is a closed chain passing through ${v}_i$. The sum of all the labels along the closed chain (called cycle sum [120]) ${\boldsymbol{s}}\!( {\boldsymbol{c}} ) = \mathop \sum \nolimits_{k = 1}^M {{\boldsymbol{l}}}_k$ equals the cell offset between the pair of equivalent atoms (in the above example, it is $0 + {{\boldsymbol{l}}}_1 - {{\boldsymbol{l}}}_0 + {{\boldsymbol{l}}}_0 - {{\boldsymbol{l}}}_2 = {{\boldsymbol{l}}}_1 - {{\boldsymbol{l}}}_2$). All the closed chains form a vector space known as cycle space and the generating subspace is composed by basic cycles. Then the dimensionality of the component X can be obtained by calculating cycle sums of basic cycles:


(4) \begin{eqnarray*} \dim \! \left( {\boldsymbol{X}} \right) = \dim \! \left( {\left\{ {{\boldsymbol{s}}\!\left( {\boldsymbol{c}} \right)|{\boldsymbol{c}} \in {\boldsymbol{F}}} \right\}} \right) = {\rm{rank}}\!\left( {\boldsymbol{S}} \right). \end{eqnarray*}

Here ${\boldsymbol{F}}$ is the set of basic cycles of the QG and ${\boldsymbol{S}}$ is a matrix containing all the basic cycle sums.

#### Molecule detection

It's worth noting that since the dimension is defined based on component X, different components in the same configuration may have different dimensionalities [121]. Considering that in molecular crystals, regardless of the dimensionality of other atoms, the molecule itself is always 0D. Therefore, using this technology [70], we can automatically extract the molecules in the molecular crystals with no need to give its prototype in advance. Treating each molecule as a block during crossover and mutation can reduce the search space and accelerate the searching process. In order to distinguish from the scheme below, we call this method **Mol-1** in this article.

Although the **Mol-1** can automatically detect molecules in molecular crystals, it is not applicable to extended systems without 0D components. To address this issue, we propose another evolution scheme, **Mol-2**, based on community detection, which can be used in extended systems. In graph theory, a community refers to a subset of nodes that are densely connected and loosely connected to nodes outside the subset in the same graph. Community detection aims to divide a graph into pieces, with few links between different parts. In this work, we utilize the Girvan-Newman (GN) algorithm [122,123] to start with the complete graph and take off the edges iteratively according to its ‘edge betweenness centrality’ (EBC). EBC is defined as the number of the shortest paths between any two nodes that pass through an edge in a graph. Those edges between two communities will have a large EBC because the shortest path between nodes in the two communities has to go through them and the edges inside a community will have a relatively small EBC. This algorithm does not provide guidance on when to stop removing edges. But for our task, it is a good choice to stop when every component is 0D since the degrees of freedom are minimized. After the decomposition process, these 0D components can be viewed as a whole and keep their connection relationship, just as molecules in the molecular crystal.

**Figure 5.**
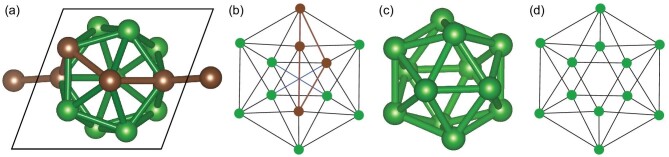
Decomposition of alpha boron. (a) The original 3D alpha boron which cannot be decomposed by **Mol-1**. The brown atoms show how one atom is extended to the equivalent atom in the other cell. The other two directions are similar. (b) The quotient graph of (a). The black edges are the bond between atoms in the same cell and the blue edges are the bonds between atoms in different cells. The brown nodes and edges are corresponding to the atoms and bonds in (a). The circle sum is (1, 0, 0) and it corresponds to the structure extended in the x-axis in (a). (c) The boron icosahedron decomposed from (a). (d) The quotient graph of (c). The edges are all black so the circle sum is all (0, 0, 0), which means (c) is a 0D cluster and the algorithm will break.

The algorithm in detail is as follows. The input to the algorithm is a QG of a crystal to be decomposed. We first calculate the dimension of each connected component. Then all 0D components are removed from the graph and added to the output list. If the graph is empty, our work is done; otherwise, we apply the GN algorithm to the remaining graph until a new 0D component is generated, and repeat this process until all components become 0D. For instance, as illustrated in Fig. 5a, alpha boron is a 3D structure, and its quotient graph is shown in Fig. 5b. The black edges represent bonds between atoms in the same cell with the label (0, 0, 0) and the blue ones are bonds between atoms in different cells with the label (1, 0, 0), (0, 1, 0) or (0, 0, 1). From this, we can see the dimension is three because the rank of cycle sums of basic cycles is 3. Next, we calculate the betweenness of each edge, and, as expected, the three blue edges have the highest value. (See online supplementary material for a color version of this figure.) After removing these three edges, the quotient graph becomes Fig. 5d, and we can easily conclude that its dimension is 0 since only (0, 0, 0) labels remain and so any cycle sum is (0, 0, 0). Therefore, the break condition has been reached, and we obtain the decomposition results shown in Fig. 5c. The boron icosahedrons and the bond connectivity are maintained in the following operators, as a result, the degrees of freedom of the searching space will be significantly reduced.

### Machine-learning accelerated local optimization

To further speed up the structure search, another important aspect is to reduce the time required for structure relaxation. One commonly used approach is to use a surrogate model to replace the computationally expensive DFT calculations. Here, we train an on-the-fly ML model during the searching process and use this model to select and relax candidate structures to accelerate global searches.

#### Machine-learning potentials

Machine-learning potential aims to find a function that takes in a configuration and directly provides its energy. To achieve this, a popular approach is to divide the total energy among the atoms proposed by Behler and Parrinello [124], this format ensures the scalability of the cell expansion, allowing MLP to be trained with configurations containing few atoms and inferred in a large system. In this model, the atomic energy is entirely determined by its local chemical environment, which is a sphere centered on the atom with a cutoff radius depending on its element type. It should preserve the symmetries of the system, including translational invariance, rotational invariance and permutational invariance. Notice that this is exactly the requirement for comparing structures using the atomic environment method. As a result, most descriptors applied in machine-learning potentials, such as atom-centered symmetry function (ACSF) [124,125]), smooth overlap of atomic positions (SOAP) [94], atomic cluster expansion (ACE) [126], and moment tensors [89] can also be used to compare structures.

Once the descriptors are determined, there are several fitting models to establish the relationship between them and atomic energy, such as linear regression, Gaussian process regression and neural network. Considering the balance between efficiency and precision [127], we use the MLIP package [90] in this work. However, some other models, such as the GPUMD package [128] are supported as well.

#### Active learning MLP in EA algorithm

The main challenge of using machine-learning potentials in structure predictions is the lack of a well-trained potential. Since the structures to be relaxed are unknown, they cannot be added to the training set in advance, and the models may give unreliable results when they encounter extrapolated atomic environments. Training a state-of-the-art general-purpose potential is not easy, it requires a lot of preparation to construct a large dataset. Although some efforts have been made to get a reasonable potential from less training data, a more feasible approach is to start with a robust potential and improve it gradually by adding new data to the training set and retraining the potential on-the-fly. This is known as active learning.

Combined with this approach, we present a new flow chart of our structure search process. The main differences from the classic evolutionary algorithm are shown in Fig. 1b. After generating random structures, we select some of them to construct the training set with DFT single-point calculations. This process may need to be repeated several times for good initialization. The most time-consuming DFT local optimization is replaced by auxiliary active machine-learning potentials. The on-the-fly process involves four parts, similar to the active learning process in MLIP package [129,90]. First, structures are relaxed using the trained potential, and extrapolation grades are estimated at every step of local optimization. The relaxation process ends correctly if all convergence criteria are met, or broken when the extrapolation grade of some structures is greater than a given threshold, indicating that the machine-learning potential is very unreliable. If the relaxation failed, we select structures from the trajectories during relaxation, calculate their properties, add them to the training set, and retrain the enlarged training set. The crucial aspect of the above process is to estimate the extrapolation grade of a structure. Several algorithms can be applied including Gaussian process regression, a neural network with drop-out layers, bragging, and D-optimality criterion. In this paper, we use the scheme of the MLIP [90,129] based on the last query strategy, which aims to construct a training set with the maximal value of the determinant of the information matrix. And the extrapolation grade is estimated by how the atomic description can enlarge the determinant of the matrix corresponding to the training set [129].

Generally, machine-learning models only provide approximate results, and a small error may cause a large change in the structures’ ranking by energy in the systems with many local minima sharing closed energy. Therefore, we should relax all potential candidate structures with relatively low enthalpies using DFT if needed. Bad fitting structures will be added to the training set to avoid waste since every DFT data is precious.

## PERFORMANCE TESTS

### Basic performance

We tested our code on three classic systems: TiO_2_, SrTiO_3_ and Mg_3_Al_2_Si_3_O_12_ which have previously been used as benchmarks in many CSP software. Due to the inherent randomness in CSP, a few tests are not enough to fully evaluate the performance of the code, and it is costly to perform many DFT calculations. Therefore, all the local relaxation calculations in the tests for these three systems are all performed 50 times by classical molecular dynamics code GULP [130] the results of which can be seen in Table 2.

**Table 2.** Basic performance test for the three systems. N_atoms_, number of atoms; N_p_, population size; N_structure_, average number of structures to find the target phase.

System	N_atoms_	N_p_	N_structure_	Success rate
TiO_2_	48	10	16	100%
SrTiO_3_	50	40	60	100%
Mg_3_Al_2_Si_3_O_12_	160	50	585	100%

To test the efficacy of our code, we began with the simplest task of predicting the ground state structure of TiO_2_ at zero pressure. We used the same empirical potential [131] as used in the previous tests [33,102]. This system has a ground state rutile with six atoms in the unit cell and various metastable phases such as anatase, brookite, hollandite and ramsdellite. The volume was generated from 344 Å^3^ to 1032 Å^3^ in the first generation and dynamically adjusted in subsequent generations. The construction was loose, as the target volume is 485 Å^3^. There were 10 individuals in one generation and the max generation was 40. The success rate was 100%, and the average generation to find the target rutile was 6.5 without cell splitting. When we added cell splitting with a factor randomly chosen from 2, 4 and 8 during the generation of random structures, the average generation fell to 1.6 with the success rate still 100%.

SrTiO_3_ with 50 atoms at zero pressure is a more complex example and was used to prove the power of an evolutionary algorithm over pure random search [132]. We use the interatomic potential proposed by Benedek *et al.* [133]. The target phase is *Pm-3m* with five atoms in its unit cell and the total volume is 600 Å^3^ for 50 atoms. The min volume and max volume are set to 388 Å^3^ and 1165 Å^3^ in the first generation. The size of each generation is 40 structures and the number of generations is 40. The success rate is 80% and the mean generation is 10.9 to get the perovskite structure. With random cell splitting ranges in 2 and 5, the results improved significantly with a 1.5 average generation and 100% success rate. These results mean the target structures can be generated at the very beginning for TiO_2_ and SrTiO_3_ with cell splitting.

We then move on to the more challenging example of Mg_3_Al_2_Si_3_O_12_. The ground state is the *Ia-3d* phase with 160 atoms in the unit cell. We used the THB model [134] to perform local optimizations. We limited the total volume to a small range from 1400 Å^3^ to 1500 Å^3^, and the maximum number of generations was 60, and 50 structures were relaxed in every generation. In our 50 times tests, an average of 585 structures are required to find the global minimum.

### Graph theory performance

**Figure 6.**
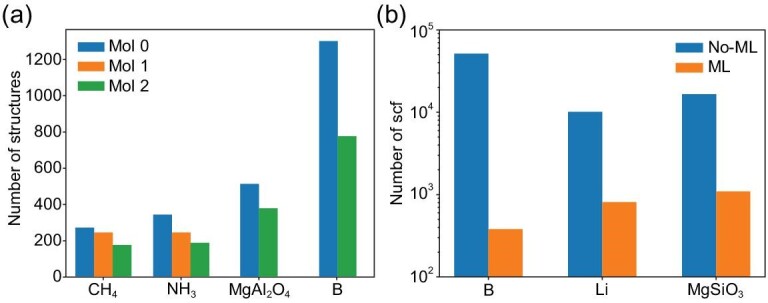
Comparison of computational efficiency. (a) Different mol algorithms; (b) without and with machine-learning acceleration.

In our recent work [70], we investigated the impact of decomposing structures using graph theory. Here we briefly review the results. In that work, we tested four systems namely methane, ammonia, boron and magnesium dialuminate for the following reasons. The $P{2}_1{2}_1{2}_1$ methane is a representative molecular crystal for which our algorithm was initially designed. The $P{2}_1/m$ phase ammonia is a mix-dimensional structure not only including isolated molecules but also chains. The gamma boron does not contain molecules but has B_12_ components and the $Pnma$ MgAl_2_O_4_ even not having obvious motifs. We tested **Mol-0, Mol-1** and **Mol-2** algorithm for the first two cases and **Mol-0, Mol-2** for the latter two, because **Mol-1** is unsuitable if structures do not contain molecules. Our results, depicted in Fig. 6a. indicate that both **Mol-1** and **Mol-2** algorithm increase the success rate from 70% to 100% for CH_4_, with **Mol-2** even displaying superior performance in the average number of attempts required to identify the global minimum. The result is similar for NH_3_. This is not surprising since we didn’t use the molecule crystal generator and many configurations may be extended after structure optimization in which case **Mol-1** works badly. For MgAl_2_O_4_ and gamma boron, **Mol-2** can also significantly reduce the number of structures required to be explored by 26% and 40%, respectively. The number to get the target MgAl_2_O_4_ (378) is comparable to the optimal results obtained using USPEX (368) [75] and CALYPSO (358) [135] as far as we know. And the gamma boron demonstrated greater progress than MgAl_2_O_4_ since boron has a more distinct community structure, and our community detector algorithm can identify them to make more constraints in the search space.

### Machine-learning performance

We have tested our machine learning based new workflow for three tasks: boron at 0 GPa, lithium at 50 GPa, and magnesium silicate at 150 GPa.

Boron is a highly complex system that has received much attention due to its complex potential energy surface. To test our method, we chose relatively simple alpha boron as a starting point, allowing us to avoid dealing with other more complex boron configurations, such as beta or tau boron. We performed both DFT and ML searches on 12-atom systems, keeping all the basic parameters the same, except for the number of individuals in each generation. Each ML-based search involved two iterations generating 4000 random structures in total, of which 241 structures were selected into the initial training set to initialize the on average potential. During the subsequent optimization process, an average of 139 structures were selected on-the-fly and calculated by DFT to find the structure very similar to the target alpha boron. The structure only differs slightly in boron-boron bond length and cell parameters, and can be further relaxed to the correct structure by DFT at the end of the search process. Thus, the machine-learning search performed 380 DFT single-point calculations, enhancing the search efficiency compared to the 51 498 single-point calculations required for a pure DFT search. After the training end, the root mean squared error (RMSE) for energy per atom, forces, and stresses were 0.04 eV, 0.23 eV/Å, and 6.5 GPa, respectively.

The boron was searched at zero pressure and then we tested our method at high pressure. Here we take lithium, the lightest metal with a lot of complex high-pressure phases, under 50 GPa as an example. At this pressure, the stable phase of lithium is *cI16* at 0 K and unknown at ∼260 K [47], which may be energetically competitive to *cI16* at zero temperature. For this purpose, we performed searches in a relatively large range of number of atoms from 4 to 40. In our tests, the potential can get the target *cI16* but with the wrong rank of enthalpy, so the DFT correction is required in each generation. Even so, only 808 DFT single-point calculations are required to find *cI16* with ML and 10 105 without it. We also found several candidates that have enthalpies close to *cI16*, one of which shows its dynamic stability at finite temperature [136]. The RMSE, at last, is 0.02 eV for energy per atom, 0.09 eV/Å for forces and 1.18 GPa for stresses.

In the final test, we aimed to demonstrate the applicability of our method to a ternary compound, specifically MgSiO_3_ at 150 GPa. MgSiO_3_ is one of the main components of the Earth's lower mantle and undergoes a transformation from the Pbnm perovskite structure to the Cmcm post-perovskite structure at ∼120 GPa [137,138]. We searched for 10–20 atoms in a unit cell and successfully found the phase both in our DFT tests and ML tests with 16 583 and 1079 DFT single-point calculations, respectively. The RMSE of the forces is relatively large (1.66 eV/Å) in the whole training set, but considering the complexity of the structure and the large range of both the enthalpy and the forces, we can accept the results since it gave the correct sort of structures.

## APPLICATIONS

Crystal structure predictions play an important role in the high-pressure field [4,5,139,140], as it can be challenging to determine the atomic structure of a synthesized phase *in situ* under extreme conditions. For example, the pressure transfer medium affects the spectroscopic detection; the metallization of many materials has a large fluorescence which hinders the Raman detection; only the low angle X-ray diffractive peaks can be observed; the samples under test exhibit a very large anisotropy and may react with the diamond anvils or pressure medium and gasket; high-pressure neutron diffraction experiments are also very difficult. Hence, we primarily concentrated on exploring the applications of our MAGUS code in the high-pressure field and achieved some interesting results [69,71,72,141–158].

### Unexpected compounds in the interior of planets

The planetary interior provides a natural laboratory for studying materials in extreme conditions and offers many useful mineral compounds and materials for mankind. Due to the lack of effective observation approaches and difficulties in experimental simulations at such high temperatures and pressures, this is where crystal structure predictions can be helpful in terms of not only the interpretation of experimental results but also the rational design of the experiment process.

**Figure 7.**
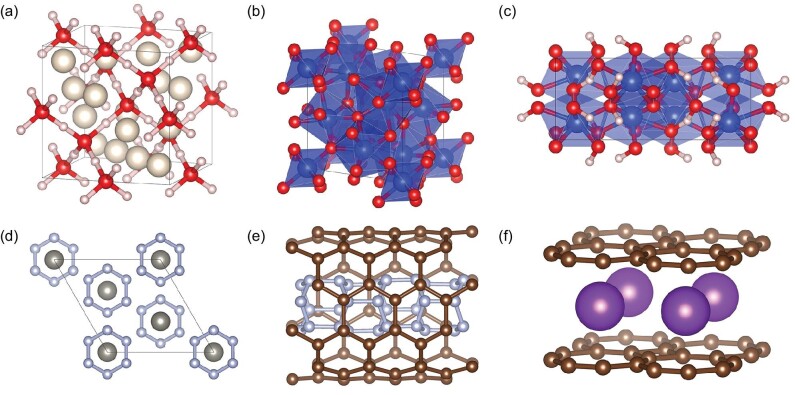
Some structures predicted by MAGUS. (a) ${\textit{Fd}}\bar{3}m{\rm{\ H}}{{\rm{e}}}_2{{\rm{H}}}_2{\rm{O}}$ [71]; (b) *R*$\bar{3}{\rm{\ Si}}{{\rm{O}}}_2$ [72]; (c) *C2c*${\rm{\ }}{{\rm{H}}}_2{\rm{O}}{( {{\rm{Si}}{{\rm{O}}}_2} )}_2$ [151]; (d) *R*$\bar{3}m{\rm{\ W}}{{\rm{N}}}_6$ [69]; (e) nitrogen nanotubes inside CNTs [152], and (f) $P4/mmm{\rm{\ }}{{\rm{C}}}_4{\rm{K}}$ [143].

Helium is the second most abundant element in the universe (just below hydrogen) and is widely dispersed throughout the atmospheres of gaseous giant planets, such as Uranus and Neptune. Recent reports suggest that helium can react with other matter under high pressure, raising questions about whether helium reacts with other matter inside giant planets. Along this line, we found a series of stable compounds of helium that reacted with the main components of the ‘hot ice’ layer of Uranus and Neptune, water [71], ammonia [146] and methane [147]. For helium-water compounds [71], we predicted two different stable stoichiometries, both of them have a superionic state under high pressure and high temperature. Interestingly, in the *Fd-3m* HeH_2_O (Fig. 7a), hydrogen atoms melt after helium within a fixed oxygen sublattice. For helium-ammonia compounds [146], we reported three new stable stoichiometries and eight new stable phases under pressures up to 500 GPa. Among them the *I4 cm* He(NH_3_)_2_ can gain its stability at ∼3 GPa and transfer to a plastic state by temperature, characterized by freely rotating ammonia molecules in a fixed nitrogen lattice. For the helium-methane system [147], we found that a He_3_CH_4_ compound is stable over a wide range of pressures from 55 to 155 GPa and a HeCH_4_ compound becomes stable around 105 GPa. Meanwhile, the *P6_3_mc* He_3_CH_4_ exhibits the coexistence of diffusive helium and plastic methane.

Silica is a crucial mineral for planetary formation and evolution, being a major component of the Earth's crust and mantle, and serving as a raw material for various industrial products. Research into new phases of silica has garnered significant interest across many fields. We predicted that a ground-state crystalline phase of silica with $R\bar{3}$ symmetry (Fig. 7b) is stable at around 645–890 GPa [72], which contains six-, eight-, and nine-coordinated silicon atoms and results in an average coordination number of eight. In addition, silica mixed with water and hydrogen were also investigated and we found a silica-water compound (SiO_2_)_2_H_2_O (Fig. 7c) can survive above 450 GPa [151]. At the conditions in the interiors of Uranus and Neptune, these compounds exhibit superionic behavior, which may have implications for the origin of anomalous magnetic fields of giant planets. When silica is mixed with helium [159], four stable phases of the HeSiO_2_ compound have been identified in the pressure range of 600–4000 GPa. These reactions between silica and helium, as well as water, may lead to the erosion of the rocky core of giant planets and form a diluted core region at this pressure.

The predictions of the exotic compounds in the interior of giant planets as well as many discoveries by others [160–163], indicate that there may exist many unexpected compounds under the extreme conditions of the interiors of giant planets. In addition, these compounds may exhibit exotic states, such as superionic, plastic, partially diffusive and their coexistence, etc. These compounds and their states may affect the structure and evolution of planets and have some important implications for the mysterious scientific questions of giant planets.

### Superhard and high-energy-density materials

MAGUS can be also applied in the exploration of new functional materials such as superhard materials and high-energy-density materials, which are important topics in high-pressure fields. Because high-pressure methods are effective ways to synthesize and explore these types of materials.

Transition metal nitrides have been suggested to have both high hardness and good thermal stability, but stable superhard transition metal nitrides are seldom synthesized. By using MAGUS, we designed a stable superhard tungsten nitride WN_6_ (Fig. 7d) which can be quenched to ambient pressure after high-pressure synthesis [69]. We estimated that the hardness of WN6 is ∼56 GPa, making it the hardest among the transition metal nitrides known so far. Additionally, the nitrogen atoms in WN_6_ form a single-bonded armchair N_6_ ring, which can release a large amount of chemical energy relative to the N≡N triple bond in nitrogen molecules. Therefore, WN_6_ is also a potentially high-energy-density material due to its good gravimetric (3.1 kJ/g) and volumetric (28.0 kJ/cm^3^) energy densities. Based on theoretical predictions and experimental efforts, the WN_6_ compound has been successfully synthesized by our experimental collaborators using the laser heated diamond anvil cell method [164].

Polymeric nitrogen also has a large potential to be a high-energy-density material for its ability to release a large amount of energy when it breaks down into nitrogen dimers. To acquire structures under mild conditions, we turned our focus to metal nitrides. We made systematic searches from metal pentazolate including bi-valence MN_10_ (M=Be, Mg, Ca and Ba) [144,150], tri-valence MN_15_ (M=Al, Ga, Sc and Y) [145], and quad-valence MN_20_(Ti, Zr) [154], to chain-linked N_4_^2−^ reactions with Al, Ga, Y and Ti at moderate pressure and temperature [153]. We also studied the polymeric nitrogen inside confined systems such as carbon nanotubes [152]. We discovered several quasi-one-dimensional single-bonded polymeric nitrogen structures, formed as nitrogen chains and tubes inside carbon nanotubes, mechanically stable at ambient pressure and may be synthesized experimentally at relatively low pressure. Two of them are composed of nitrogen tubes (Fig. 7e). Calculations show all the structures above will release a lot of energy sometimes even larger than TNT and HMX during the explosion process, thus they can be used as potential high-energy-density materials.

### Superconducting materials

Superconducting materials can be widely used in many fields such as particle accelerators, levitating, magnetic resonance imaging (MRI), etc. The search for new superconductors has been a perennial hot topic for condensed matter physics and materials science.

Single-layer superconductors are ideal materials for fabricating superconducting nanodevices. T-graphene, a single-layer planar carbon sheet with 4- and 8-membered rings, is a new intrinsic elemental superconductor. We proposed a synthesis route to obtain such a single-layer T-graphene [143], that is, a T-graphene potassium intercalation compound is first synthesized at high pressure (>11.5 GPa) and then quenched to ambient condition; and finally, the single-layer T-graphene can be either exfoliated using the electrochemical method from the bulk C_4_K (Fig. 7f) or peeled off from bulk T-graphite C_4_, where C_4_ can be obtained from C_4_K by evaporating the potassium atoms. Interestingly, we found that the calculated *Tc* of C4K is ∼30.4 K at 0 GPa, which sets a new record for layered carbon-based superconductors.

Very recently, we predicted that lithium and aluminum can form stable alloys under high pressure with interesting properties [156]. For instance, in Li_2_Al and Li_3_Al_2_, we found dimensional reductions of electronic structures with significant step-like features near the bottom of the valence band. In the three Li-rich compounds (Li_3_Al, Li_4_Al, Li_6_Al), both superconducting and superionic behaviors occur in the same system, at different temperatures, which reflect the transport properties of two kinds of particles with very different masses, electrons and ions.

## CONCLUSION

We developed a crystal structure predictor MAGUS accelerated by graph theory and machine learning. It can not only work in bulk crystals [71,72,141,142,146,147,149,151,156], but also many other systems including clusters, surfaces [105], 2D crystals [143,148] and molecular crystals [144,145,150,154], as well as confined systems [152]. By default, or user-defined fitness function, it can predict stable chemical compositions among stoichiometric space or explore metastable structures with target properties. The details in our basic version including random structure generation, variation operators and structure comparison are described. We interpret the special features of molecular crystals and how to take the space group of the molecule into account. The effect of cell splitting is shown and can help improve the results when the crystal structure to predict is large and complex. With structure fingerprint, the clustering method was applied to help keep the balance between quality and diversity. For graph theory, we have explained the basic idea of a quotient graph for crystal and discussed two novel variation schemes based on dimension identification and community detection. For machine learning, we briefly introduced the development of machine-learning potential and how to train a potential on-the-fly from scratch. The new workflow in combination with active learning is given and three new parts including preparation for the initial dataset, on-the-fly machine learning accelerated relaxation, and DFT check is presented.

We performed benchmark calculations of our MAGUS code in TiO_2_, SrTiO_3_ and Mg_3_Al_2_Si_3_O_12_, and the results are competitive with other popular softwares. The improvement of graph theory and machine learning are tested in different systems and we demonstrated that they are very useful. With graph theory and machine learning, the success rates are increased and the DFT single-point calculations can be reduced a lot, which substantially enhanced the search efficiency. The successful examples achieved by MAGUS are shown in different fields, including unexpected compounds in the interior of planets, functional materials with superhard, high-energy density, and superconducting properties, etc. For planetary science, we study how the abundant elements in the giant planets may mix under high pressure and help to understand the planetary models. Particularly, if the unexpected compounds we predicted can be verified by high-pressure experiments in the future, these compounds could provide reliable data (such as density, elastic and conductive properties, etc.) for building planetary models. This will affect the structure and evolution of planets and help to explore a new research paradigm of planetary science, starting from theoretical predictions based on quantum mechanics, to experimental verifications and then further applications in planetary models. For high-energy-density materials, we studied nitride and discover a series of potential high-energy-density materials comparable with TNT and HMX, including MN_10_ (Be, Mg, Ca and Ba), MN_15_ (Al, Ga, Sc and Y), MN_20_ (Ti, Zr), using metals with different valence. For superconducting materials, we studied the superconducting phase transitions induced by pressure, and predict a new synthetic path of T-graphene.

We demonstrate that our MAGUS code can facilitate the discovery of new materials, as well as the elucidation of the mysterious scientific questions under extreme conditions that are difficult to explore with experiments at the stage of current techniques.

## DATA AVAILABILITY

MAGUS source code can be accessed after registration: https://www.wjx.top/vm/m5eWS0X.aspx. More information can be obtained from the corresponding author of this paper.
